# Split-cube-resonator-based metamaterials for polarization-selective asymmetric perfect absorption

**DOI:** 10.1038/s41598-020-74221-7

**Published:** 2020-10-19

**Authors:** Odysseas Tsilipakos, Angelos Xomalis, George Kenanakis, Maria Farsari, Costas M. Soukoulis, Eleftherios N. Economou, Maria Kafesaki

**Affiliations:** 1 Institute of Electronic Structure and Laser, Foundation for Research and Technology-Hellas, 70013 Heraklion, Crete Greece; 2 NanoPhotonics Centre, Cavendish Laboratory, Department of Physics, University of Cambridge, JJ Thompson Avenue, Cambridge, CB3 0HE UK; 3 Ames Laboratory-US DOE and Department of Physics and Astronomy, Iowa State University, Ames, Iowa 50011 USA; 4 Department of Physics, University of Crete, 70013 Heraklion, Crete Greece; 5 Department of Materials Science and Technology, University of Crete, 70013 Heraklion, Crete Greece

**Keywords:** Optics and photonics, Terahertz optics

## Abstract

A split-cube-resonator-based metamaterial structure that can act as a polarization- and direction-selective perfect absorber for the infrared region is theoretically and experimentally demonstrated. The structure, fabricated by direct laser writing and electroless silver plating, is comprised of four layers of conductively-coupled split-cube magnetic resonators, appropriately rotated to each other to bestow the desired electromagnetic properties. We show narrowband polarization-selective perfect absorption when the structure is illuminated from one side; the situation is reversed when illuminating from the other side, with the orthogonal linear polarization being absorbed. The absorption peak can be tuned in a wide frequency range by a sparser or denser arrangement of the split cube resonators, allowing to cover the entire atmospheric transparency window. The proposed metamaterial structure can find applications in polarization-selective thermal emission at the IR atmospheric transparency window for radiative cooling, in cost-effective infrared sensing devices, and in narrowband filters and linear polarizers in reflection mode.

## Introduction

Optical systems at the mid-infrared frequency range suffer from high fabrication cost and low stability at room temperature (mostly attributed to moisture sensitivity). Spectroscopy at such frequency ranges is one of the emerging technological directions due to the atmospheric window 8–13 μm (23–37 THz). Conventionally, optical components (polarizers, waveplates etc.) used for mid-infrared spectroscopy are bulky so as to achieve strong light matter-interaction for efficient manipulation of electromagnetic waves. Metamaterials (MMs), man-made nanostructured materials composed of subwavelength building blocks (meta-atoms) in a periodic formation, offer novel opportunities for designing optical components. Harnessing the resonant nature of their constituent components, MMs allow for strong light-matter interaction over short spatial scales and can deliver atypical material properties beyond natural materials. Importantly, the obtained material properties can be tailored by tuning the resonant response of the meta-atoms via their exact geometry and material composition. The resulting component designs can be scaled to work in different parts of the electromagnetic spectrum (due to the invariability of Maxwell’s equations), especially from RF and microwave to mid-infrared frequencies where metals behave predominantly as perfect conductors. Such artificial structured materials have been proposed for various applications, ranging from polarization resolved spectroscopy and nonlinear effects to light modulators and absorbers^1–5^.

Thin layers of metamaterials, frequently termed metasurfaces (MSs), transfer the functionality of metamaterials into subwavelength thickness structures. At the same time, MSs help in minimizing Ohmic loss due to the short interaction volume. An abundance of interesting functionalities with such artificial sheet materials has been demonstrated at frequencies ranging from the microwave to the infrared and optical bands^6–8^. For example, MSs have been used to tailor the absorption and thermal emission properties^9,10^, manipulate the wavefront^11,12^, and engineer the dispersion of the induced phase^13^. Recently, metasurface structures that perform polarization- and direction-sensitive (forward or backward illumination) operations have attracted considerable attention^14–19^ due to the rich physics and new application opportunities they can provide. An invaluable asset for the physical implementation of such advanced functionalities are meta-atoms with three-dimensional geometry that can lead to polarization- and direction-selective excitation of electric or magnetic dipolar responses. For example, such meta-atoms have been exploited to achieve high asymmetry in transmitted or reflected electromagnetic waves for only one direction of illumination^20^. Asymmetric optical properties have been demonstrated for linear^20^ as well as circular polarization of light^21^. Fabricating meta-atoms with volumetric complexity and sub-micron geometric features can be a challenging task. Recently, laser printable 3D MMs have been realised with Direct Laser Writing (DLW), resulting in cost-effective and high-resolution fabrication, essential for practical applications^22^. DLW has been employed to successfully construct multi-layered periodic structures (photonic crystals and metamaterials) composed of meta-atoms with practically arbitrary geometry^23,24^. Both dielectric and metallic structures can be realized, by selectively covering polymeric scaffolds with metal nanoparticles (Ag/Au) via electroless plating^23,25,26^.

In this work, exploiting the powerful technique of multiphoton direct laser writing, we experimentally demonstrate a polarization- and direction- selective metamaterial component that can act as an asymmetric perfect absorber and linear polarizer in the less-explored mid-infrared frequency regime. The metamaterial is comprised of four layers of conductively-coupled split-cube magnetic resonators appropriately rotated to each other to: (i) support a high-quality-factor magnetic resonance, (ii) create a geometric asymmetry with respect to the structure mid-plane along the propagation direction, essential for bestowing directional sensitivity, and (iii) suppress the generation of cross-polarized reflection and transmission components. Our conductive printable micro-structure shows narrowband polarization-selective perfect (100%) absorption centered at 27 THz; the situation is reversed when illuminated from the opposite side with the orthogonal linear polarization being absorbed. The resonant frequency of the metamaterial structure can be tuned across the entire atmospheric transparency window 23–37 THz, by properly varying the geometrical parameters. In addition, the structure shows angle-independent performance for the TM polarization. Excellent agreement between theoretical predictions and measurements conducted by Fourier-Transform Infrared Spectroscopy is found, verifying the practical application perspective of our approach.

## Results

### Metamaterial structure and electromagnetic response

**Figure 1:**
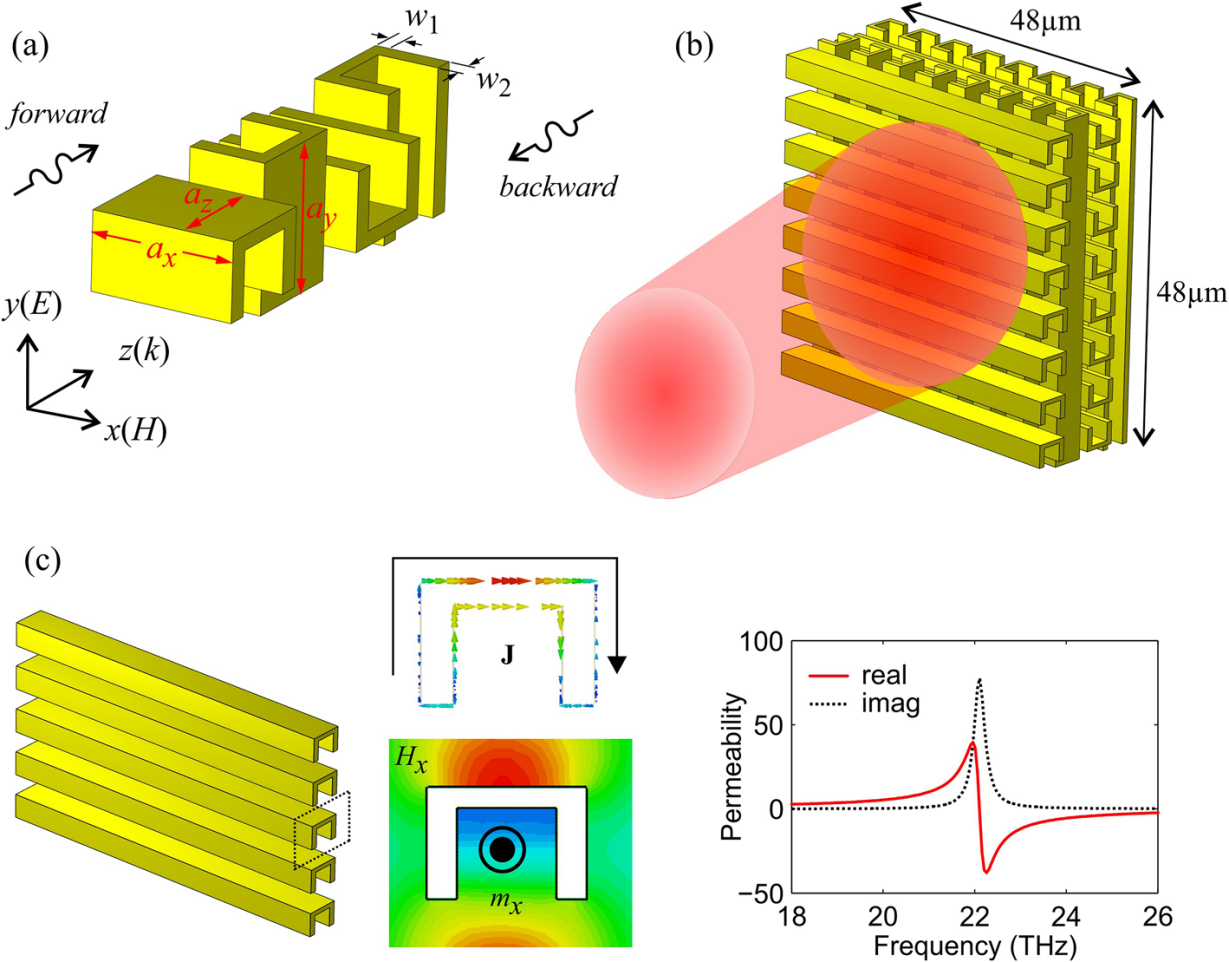
Proposed split-cube resonator metamaterial: (**a**) Four-layer unit cell with geometrical parameters. (**b**) $8\times 8$-cell structure ($48\ \mu {\mathrm{m}} ~\times\ 48\ \mu {\mathrm{m}}$) able to accommodate the incident beam in a typical experimental study. (**c**) Single row of split cube resonator metamaterial. A magnetic dipole resonance is supported around 22 THz as seen by surface current (${\mathbf {J}}$) and magnetic field ($H_x$) distributions and verified by the retrieved effective permeability.

The unit cell of our metamaterial consists of four metallic split-cube resonators (SCRs), as shown in Fig. 1a. The SCRs are rotated by 90 degrees (clockwise) with each other along the *z* propagation axis to form the four-layer structure with the desired properties, as will be discussed below. Regarding the geometric dimensions of the SCR, for perfect absorption operation near 30 THz, micron and sub-micron scale structures are required. In our case, the design procedure results in the parameters $a_x=6~\mu \mathrm {m}$, $a_y=6~\mu \mathrm {m}$, $a_z=4.55~\mu \mathrm {m}$, $w_1=0.6~\mu \mathrm {m}$, $w_2=0.85~\mu \mathrm {m}$. For an experimental demonstration of the electromagnetic (EM) response of the structure, the sample should be adequately wide to accommodate the incidence beam. Such a structure, made of $8\times 8$ unit cells ($48~\mu {\mathrm{m}} ~\times\ 48\ \mu {\mathrm{m}}$) along the two periodic dimensions, is depicted in Fig. 1b. When excited with a magnetic field parallel to its axis (${\mathbf {H}}=H_x\hat{{\mathbf {x}}}$), the split cube resonator supports a strong magnetic resonance with an induced dipole moment ($m_x$) formed by the loop-like conduction currents, as shown in the inset of Fig. 1c. The magnetic nature of the resonance is verified by the retrieved effective magnetic permeability; for the chosen dimensions, the resonant frequency for the single row/layer of SCRs appears at 22 THz. Conductive coupling enables the currents to couple between successive SCR layers, shaping the collective behavior of the composite (4-layer) system.

**Figure 2:**
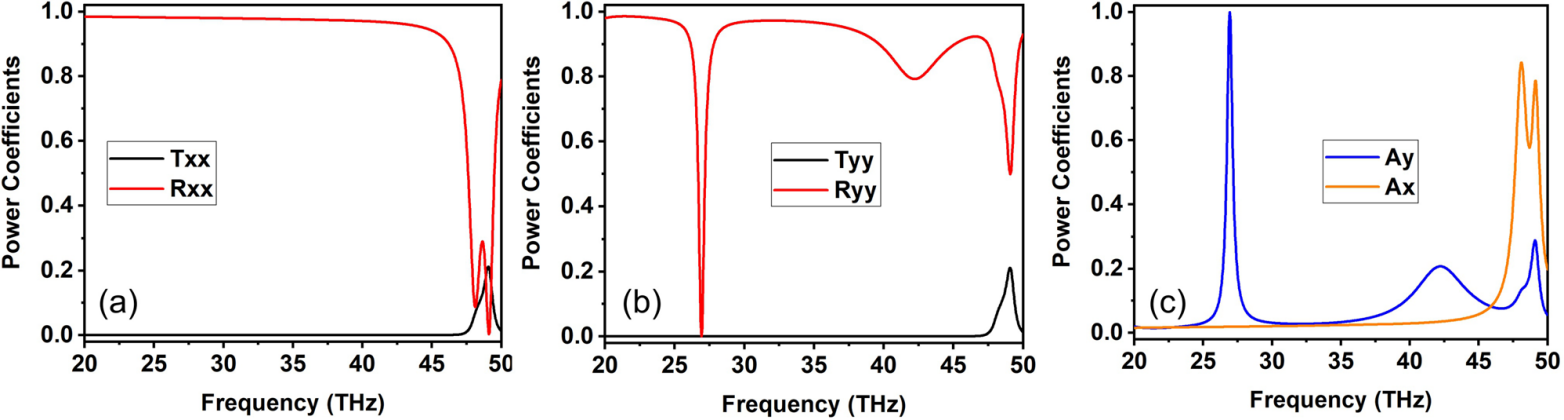
Simulation results for the four-layer SCR structure depicted in Fig. 1a with $a_x=6~\mu \mathrm {m}$, $a_y=6~\mu \mathrm {m}$, $a_z=4.55~\mu \mathrm {m}$, $w_1=0.6~\mu \mathrm {m}$, $w_2=0.85~\mu \mathrm {m}$ under forward illumination. (**a**) Reflection and transmission power coefficients for *x* linear polarization incidence (only co-polarized components are shown, since the cross-polarized ones are almost zero). (**b**) Reflection and transmission power coefficients for *y* linear polarization incidence (again cross-polarized components are vanishing). (**c**) Absorption coefficients for the two linear polarizations. The structure exhibits narrowband perfect absorption around 27 THz for the *y* linear polarization, whereas the *x* linear polarization is reflected. The situation is exactly reversed for illuminations from the backward direction (see Fig. S1 of the Supplementary Information document).

For the theoretical/numerical investigation of the structure full wave simulations are performed employing a commercial three-dimensional electromagnetic solver (CST Studio). The simulations are performed on a single unit cell with periodic boundary conditions along the *x*- and *y*- directions [Fig. 1a] and assuming for the SCRs conductivity $\sigma _{\mathrm{Ag}}=5.7\times 10^7$ S/m. The full-wave simulation results for forward illumination are depicted in Fig. 2, where we examine normal incidence for two linear polarizations (*x*- and *y*- polarized). Plane-wave reflection (*R*), transmission (*T*), and absorption (*A*) power coefficients are plotted in a spectral range up to 50 THz; for higher frequencies, higher diffraction orders become propagating since the periodicity ($a_x=a_y=6~\mu \mathrm {m}$) starts to exceed the free space wavelength ($\lambda _0=6\ \mu$m at 50 THz). In the plotted transmission and reflection, the first (second) subscript denotes the polarization of the output (input) wave. For example, $R_{yx}^{fw}=|E_y^r/E_x^i|^2$, where $E_y^r$ stands for the reflected *y*-polarised electric field and $E_x^i$ stands for the incident *x*-polarised electric field; the superscript “*fw*” stands for “forward”. The absorption is calculated for each polarization and incidence direction through $A_j^{fw}=1-\sum _i R_{ij}^{fw}-\sum _i T_{ij}^{fw}$, where $i,j=\{x,y\}$. The calculated absorption is also verified by integrating the power loss density in the structure, yielding identical results.

Focusing in the spectral regime near 30 THz falling in the atmospheric transparency window, the structure for the *y* linear incident polarization exhibits a narrow “perfect” absorption peak near 27 THz [Fig. 2c]. This perfect absorption peak leads to a corresponding zeroing of $R_{yy}^{fw}$, as seen in Fig. 2b. On the other hand, the *x* polarization is simply reflected at these frequencies [Fig. 2a]. This is because the *y* linear polarization succeeds in exciting the magnetic dipole resonance via the $H_x$ component, which is parallel to the induced magnetic dipole moment in the first/front layer of SCRs [cf. Fig. 1]. In contrast, the *x* linear polarization cannot excite the magnetic dipole resonance, due to the orientation of the exciting magnetic field which does not allow coupling with the front SCR layer; the front layer behaves in that case as a grid of uniform conducting wires, impeding the coupling of the incident field with the other (inner) SCR layers and leading to almost total reflection. Note that the frequency of the magnetic dipole resonance has shifted in the 4-layer SCR structure as compared with the single-layer SCR structure from 22 to 27 THz, a result of conductive coupling with the other SCR layers. The situation is exactly reversed for the backward illumination direction, as can be easily identified via geometric considerations, with the *x* linear polarization being capable of exciting the magnetic dipole resonance and, thus, being perfectly absorbed, and the *y* linear polarization being reflected (see Fig. S1 of the Supplementary Information document). The asymmetry in absorption for the two directions of incidence reaches almost 100% [Fig.2c].

**Figure 3:**
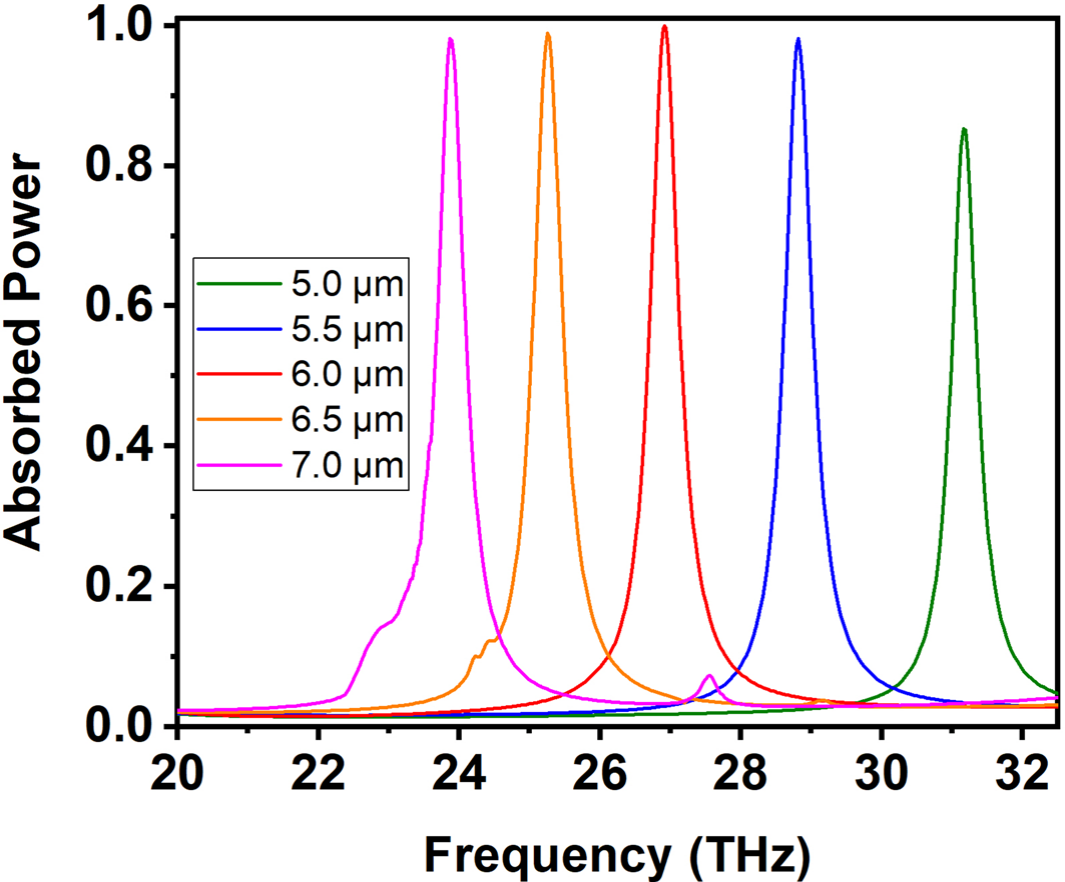
Absorption spectrum for varying unit cell size: $a_x=a_y=\{5,5.5,6,6.5,7\}~\mu$m, i.e., a denser or sparser packing of the SCRs inside the *xy* plane. The spectral position of the sharp absorption peak can be readily tuned between 24 and 31 THz, while maintaining strong absorption performance.

The cross-polarised reflection and transmission components are zero, as will be verified in Fig. 6c,d, and are thus not shown in Fig. 2. The four-layer SCR structure succeeds in zeroing-out the cross-polarized reflected and transmission components by virtue of the selected orientations for the four SCR layers. In our previous work, we have demonstrated that two SCRs layers rotated by 90 degrees (as, e.g., the two front layers of Fig. 1b) result in the excitation of cross-polarized components, as conductive coupling between the two SCRs allows an orthogonal $m_y$ magnetic dipole moment to be induced in the second SCR layer for an *x*-polarized incident magnetic field (in addition to $m_x$ in the first SCR layer)^20^, “radiating” the cross-polarized field. In addition, the four-layer structure establishes a high-quality-factor magnetic dipole resonance resulting in a sharp absorption peak, something not possible with a single SCR layer. Finally, it allows for bestowing direction-sensitive response by breaking the mirror symmetry with respect to the *xy* plane bisecting the structure. Importantly, the actual spectral position of this sharp absorption peak can be tuned inside the entire atmospheric window by modifying the geometric parameters of the SCR, a distinct advantage of metamaterial structures that allow to tailor the effective material properties by tuning the structure geometry. Specifically, by packing the same SCRs in a denser or sparser arrangement inside the *xy* plane, changing the unit cell size in the range $a_x=a_y=\{5,5.5,6,6.5,7\}~\mu$m, the absorption peak can be continuously tuned between 24 and 31 THz, as illustrated in Fig. 3. The absorption remains high in all cases.

**Figure 4:**
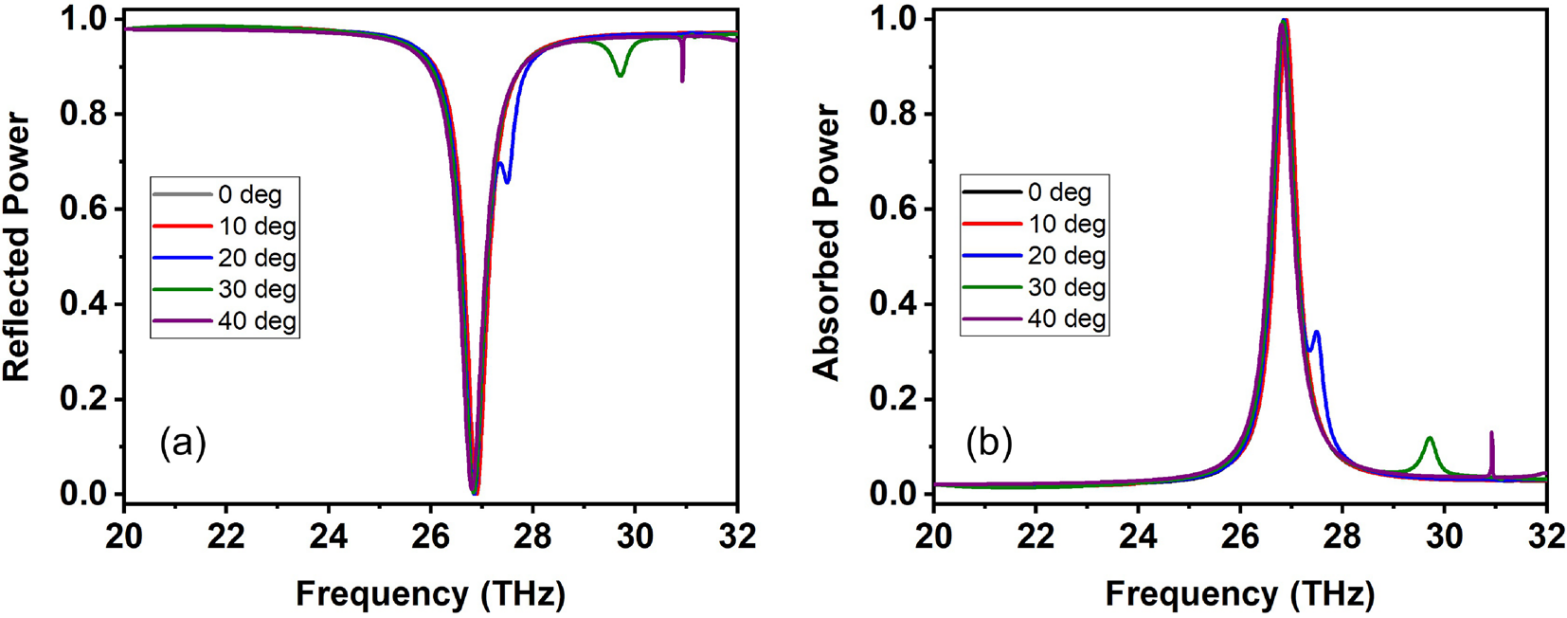
Oblique incidence performance for TM polarization in the *yz* incidence plane (${\mathbf {H}}=H_x\hat{{\mathbf {x}}}$). Reflection and absorption spectra for different incidence angles: $\theta =\{0, 10, 20, 30, 40\}$ degrees. The performance is independent of the incidence angle.

Next, we study the performance of the proposed absorber structure under oblique incidence. The results are depicted in Fig. 4 for TM polarized incidence inside the *yz* incidence plane (${\mathbf {H}}=H_x\hat{{\mathbf {x}}}$). As can be seen, the performance is practically independent of the incidence angle for angles up to 40 degrees, resulting in a broad angular bandwidth that is an advantageous trait for perfect absorbers^27^. For angles exceeding 50 degrees the absorption spectrum is disturbed by the higher-order diffraction channels that have become propagating and approach the vicinity of the main absorption peak at 27 THz; according to standard diffraction grating theory for an incidence angle of 50 degrees, the first diffraction order becomes propagating for frequencies above 28.3 THz as found from $\lambda =a(\sin \theta +1)$ given the lattice constant $a=6~\mu$m. Secondary absorption peaks appearing in Fig. 4b at frequencies above 27 THz are shallow and do not interfere with the narrowband perfect absorption behavior. The angle independence for the TM polarization is because the *x* component of the magnetic field remains constant for any incidence angle; it is exactly this component that mediates the excitation of the magnetic dipole resonance and is, thus, responsible for the in-coupling of the wave into the structure and the observed absorption peak. For TE polarized incidence in the *xz* incidence plane (${\mathbf {E}}=E_y\hat{{\mathbf {y}}}$), the magnetic field component does not remain parallel to the *x* axis and the spectral position of the absorption peak changes with incidence angle, as anticipated (see Fig. S3 of the Supplementary Information document).

### Experimental results

**Figure 5:**
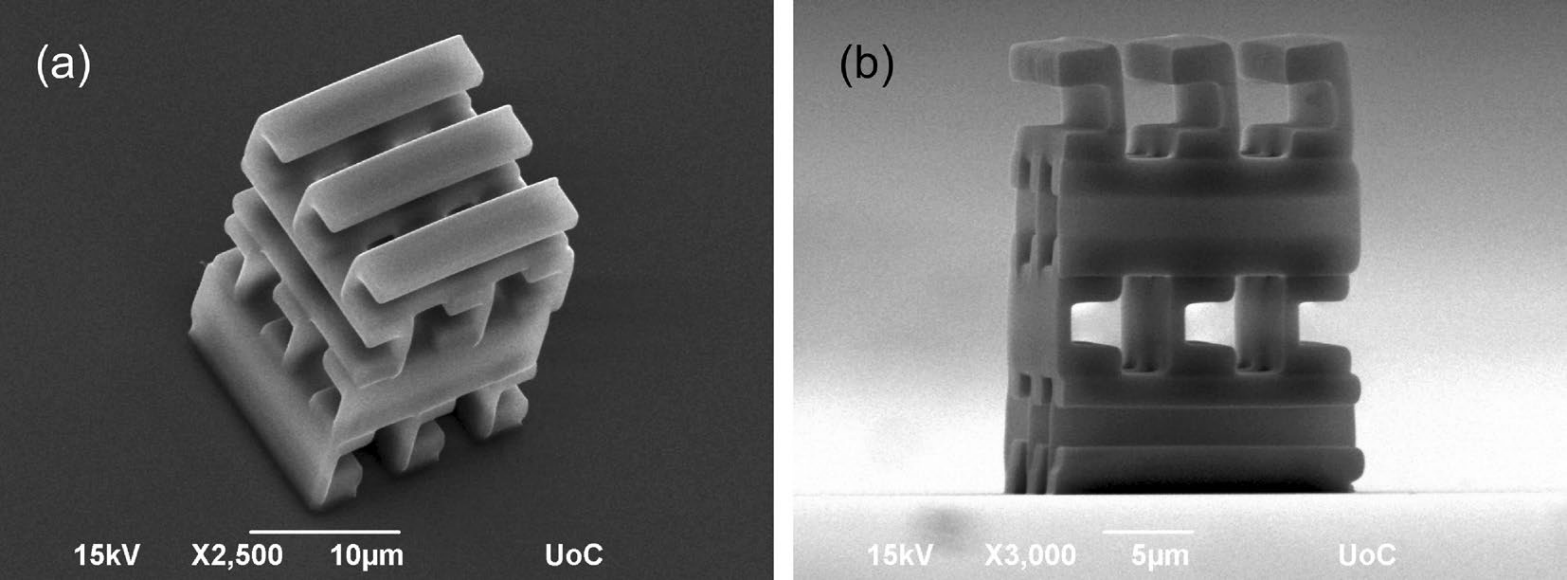
Scanning electron microscope images of the 3D four-layer SCR metamaterial structure (**a**) bird’s eye and (**b**) side view recorded at 15 kV. Scale bar is 10 and 5 μm for (**a**) and (**b**), respectively.

Next, the proposed structure was fabricated and measured with Fourier-transform infrared spectroscopy (FTIR). Samples of  $8\times 8$ unit-cells ($48~\mu$m$~\times\ 48\ \mu$m) were fabricated in order to accommodate the spot size which is approximately 40 $\mu$m in diameter. In Fig. 5 scanning electron microscope images of $3\times 3$ unit cell SCRs, which were fabricated for imaging purposes, are depicted, showing good quality and topology free from errors.

**Figure 6:**
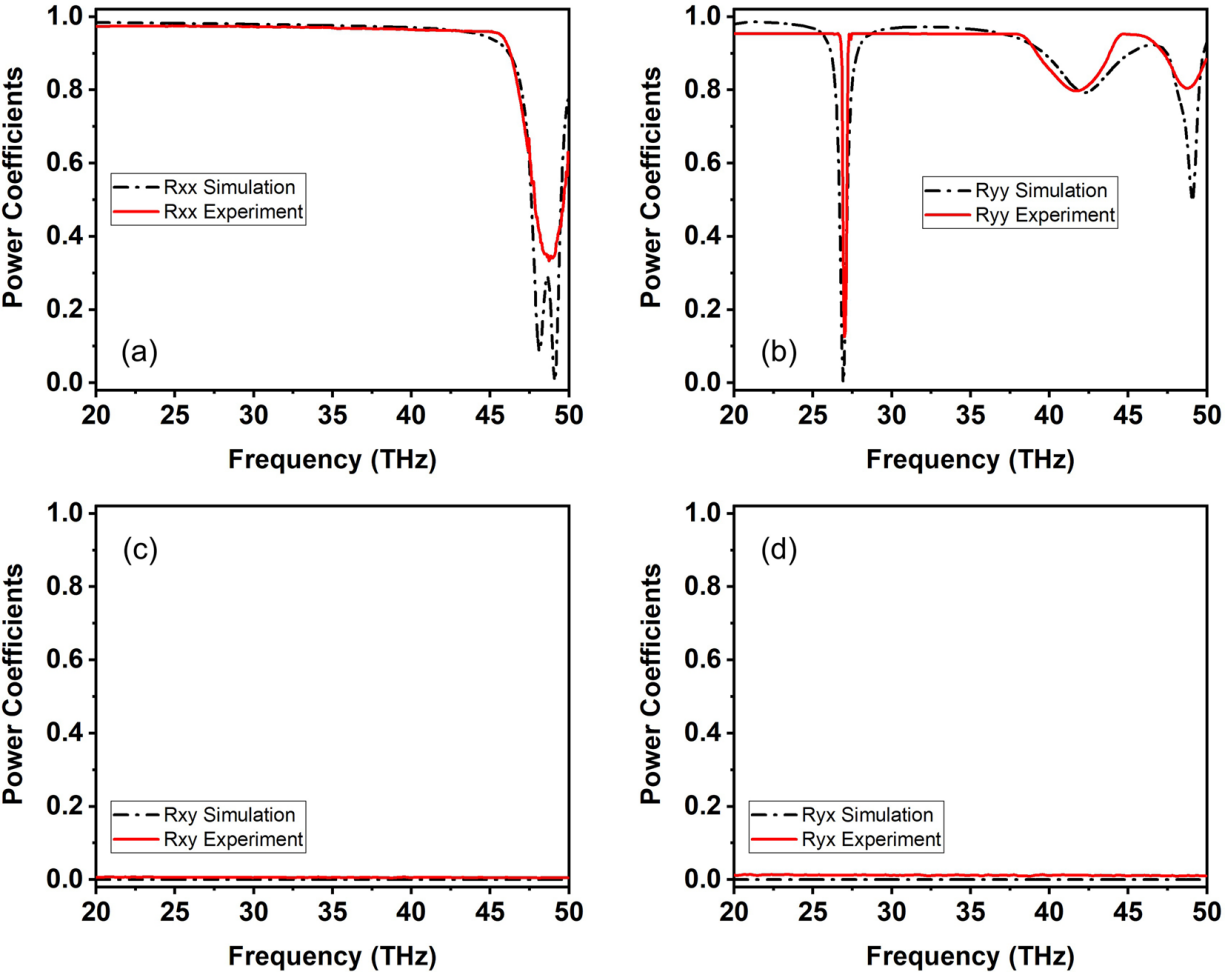
Co- and cross-polarized power reflection coefficients for a four-layer SCR metamaterial structure with dimensions $a_x=6~\mu$m, $a_y=6~\mu$m, $a_z=4.55~\mu$m, $w_1=0.6~\mu$m and $w_2=0.85~\mu$m. Comparison between experiment (solid lines) and theoretical predictions (dashed lines) showing excellent agreement.

The fabricated MM structure resides on a cover slip which is approximately 1-mm thick precluding transmission. As a result, in the experiment only the forward illumination direction is examined and thus comparison with the theoretical results calculated for the free-standing (no substrate) structure is performed on the co- and cross-polarized reflection components. We have verified via simulations that a presence of a $\hbox {SiO}_2$ substrate does not alter the reflection coefficients under forward illumination, permitting such a comparison. This is because the electromagnetic wave is either reflected from the input of the four-layer structure (*x* polarization) or absorbed within it (*y* polarization), but without reaching the substrate to interact with it, as can be seen in Fig. S2 of the Supplementary Information document which depicts the induced surface current density at 27 THz for both *x* and *y* polarizations. The experimental and theoretical results are depicted together in Fig. 6; experimental data for the co- and cross-polarized reflection power coefficients are plotted with solid lines and simulation results with dashed lines. In all cases, excellent agreement is found verifying the sharp spectral feature at 27 THz, as well as the additional features at higher frequencies. The measured dip near 27 THz is, surprisingly, somewhat sharper than the simulated response. We think that the main reason for this is due to variations of the geometrical parameters in the measured sample from the nominal values that render the resonance at 27 GHz “darker”, i.e., less radiative; to support this explanation we have verified by simulations that increasing the length $a_z$ for example [see Fig. 1a] renders the resonance darker and the associated dip sharper. Moreover, an additional factor that affects the comparison of measurement vs. simulation is the fact that in the simulation an infinite periodic structure is considered with a plane-wave excitation, whereas the experiment is conducted with a finite-size sample ($8\times 8$ unit cells) and a finite-size beam (40-μm diameter). Finally, note that the symmetry of the structure implies the absence of cross-polarized components for *x* and *y* linearly polarized illumination. This expected feature is not only confirmed by simulation results, but also experimentally verified [Fig. 6c,d], indicating that the fabrication process has preserved the nominal symmetries, thus further confirming the high quality of the sample.

## Discussion

We have studied theoretically and experimentally split-cube-resonator (SCR) based laser-printed metamaterial structures that can act as a polarization- and direction- selective perfect absorbers in the infrared atmospheric transparency window. We have measured polarization-selective perfect absorption at a frequency of 27 THz by FTIR measurements on $48\ \mu {\mathrm{m}} ~\times\ 48\ \mu {\mathrm{m}}$ fabricated samples, in excellent agreement with the theoretical predictions from full wave simulations. The actual spectral position of the absorption peak can be tuned in a broad range by simply packing the same SCRs in a denser or sparser arrangement. Along with the angle-independent response for TM polarization, these traits are appealing for practical applications. Our structures can implement cost-effective narrowband linear polarizers for the infrared, assist polarization resolved spectroscopic techniques within the atmospheric transparency window, and find use as infrared sensing devices.

## Methods

### Fabrication details

The structure has been fabricated by the multiphoton Direct Laser Writing (DLW) technique followed by electroless silver plating. DLW is a printing technique that allows fabrication of 3D structures with resolution less than 100 nm. In DLW, the beam of a pulsed femtosecond laser, Ti:Sapphire (Femtolasers Fusion, 800 nm wavelength, repetition rate 80 MHz) is focused inside the volume of a photosensitive and transparent composite leading to absorption of two or more photons resulting in well-defined localized polymerization. The laser beam focus can be positioned freely inside the three-dimensional photopolymer volume, enabling the printing of arbitrary 3D structures with a high degree of precision. The laser beam is focused through a high numerical aperture (N.A.) microscope objective (100$\times$ with N.A. = 1.4, Zeiss Plan Apochromat). The photosensitive material employed in the present fabrication is an organic-inorganic composite consisting of Methacryloxypropyl Trimethoxysilane (MAPTMS), Zirconium n-Propoxide, 2-(dimethylamino)ethyl methacrylate as a metal-binding moiety, and (4,4-bis(diethylamino) benzophone) as a photo-initiator. The material preparation, scaffold structure fabrication and subsequent silver metallization has been described in detail in Ref.^24^. Note that our plating process is highly selective allowing to metallize only the polymeric matrices.

### Measurement details

For accessing the desired frequency ranges, we performed measurements using a Fourier-transform infrared spectrometer (Bruker Vertex 70v) attached to an infrared microscope (Bruker Hyperion 2000). In order to resolve the polarization of input and output light beams we used a set of linear ZnSe grid polarizers. In addition, in our measurements we used microscope apertures of $\sim 40~\mu$m diameter comparable to the structure’s size ($\sim 48~\mu {\mathrm{m}}\times\ 48\ \mu {\mathrm{m}}$) to accommodate the beam exclusively on the metamaterial structure and exclude reflection contributions from the glass substrate.

## Supplementary information

Supplementary information
