# Supplementary material for: Split-cube-resonator-based metamaterials for polarization-selective asymmetric perfect absorption
Source: Sci Rep. 2020 Oct 19;10:17653. doi: 10.1038/s41598-020-74221-7 (PMC7572556; doi:10.1038/s41598-020-74221-7)
Supplement: Supplementary file 1 — Supplementary information [file 41598_2020_74221_MOESM1_ESM.pdf]

Supplementary Information for

Split-cube-resonator-based metamaterials for polarization-selective asymmetric perfect absorption

Odysseas Tsilipakos,[1,*,+] Angelos Xomalis,[2,+] George Kenanakis,[1] Maria Farsari,[1] Costas M. Soukoulis,[1,3] Eleftherios N. Economou,[1,4] Maria Kafesaki[1,5]

[1] Institute of Electronic Structure and Laser, Foundation for Research and Technology-Hellas, 70013 Heraklion, Crete, Greece
[2] NanoPhotonics Centre, Cavendish Laboratory, Department of Physics, JJ Thompson Avenue, University of Cambridge, Cambridge, CB3 0HE, United Kingdom
[3] Ames Laboratory-US DOE and Department of Physics and Astronomy, Iowa State University, Ames, Iowa 50011, United States
[4] Department of Physics, University of Crete, 70013 Heraklion, Crete, Greece
[5] Department of Materials Science and Technology, University of Crete, 70013 Heraklion, Crete, Greece
* otsilipakos@iesl.forth.gr
+ these authors contributed equally to this work

Document Information: This supporting information document is 4 pages long and includes 3 figures. It is structured in 3 sections:

S1. Simulation results for backward illumination of free-standing structure

S2. Field plots of perfect absorption (y linear polarization) vs perfect reflection (x linear polarization) at 27 THz

S3. Oblique incidence results for TE polarization in *xz* plane

S1. Simulation results for backward illumination of free-standing structure

In the free-standing four-layer SCR structure, forward and backward illumination directions exhibit "reversed" responses for the two polarizations, i.e., the response for the *y* linear polarization for forward illumination is identical with the response for *x* linear polarization under backward illumination and vice versa. This is shown in Fig. S1 compiling amplitude reflection and transmission coefficients, as well as the absorption, for all cases.

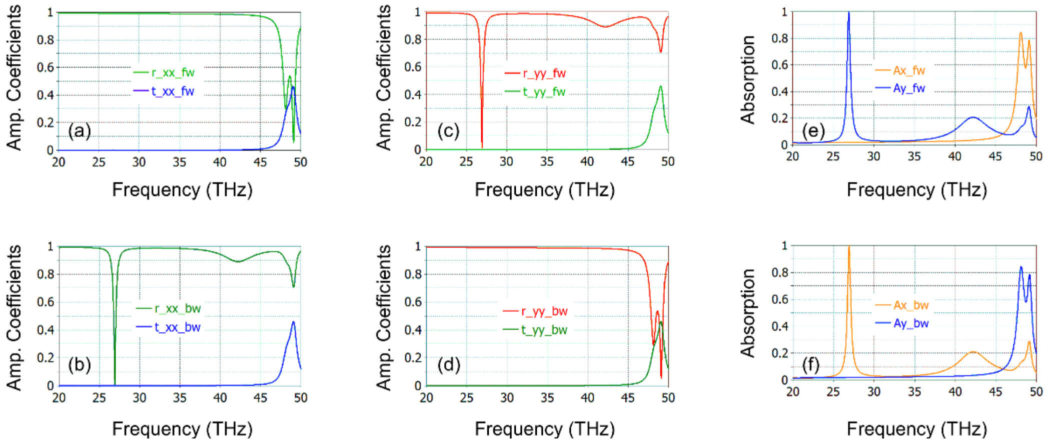

Fig. S1: Simulation results for a free-standing (no substrate) four-layer SCR structure. (a,b) Amplitude reflection and transmission coefficients for the *x* linear polarization, considering both (a) forward (fw) and (b) backward (bw) illumination. (c,d) Amplitude reflection and transmission coefficients for the *y* linear polarization, considering both (c) forward (fw) and (d) backward (bw) illumination. (e,f) Absorption for (e) forward (fw) and (f) backward (bw) illumination. The situation is exactly reversed between the two illumination directions.

S2. Field plots of perfect absorption (y linear polarization) vs perfect reflection (x linear polarization) at 27 THz

Fig. S2 depicts the distribution of the surface current density for both polarizations. For E_y excitation [Fig. S2(a)], the field penetrates and interacts with the structure, since the incident H_x field component can couple with the magnetic dipole resonance of the SCR resonators in the first/front layer. The induced current distribution then conductively couples to the other layers. In contrast, the Ex excitation [Fig. S2(b)] cannot excite the magnetic dipole resonance; the front layer behaves in that case as a grid of uniform conducting wires, impeding the coupling of the incident field with the other (inner) SCR layers and leading to almost total reflection.

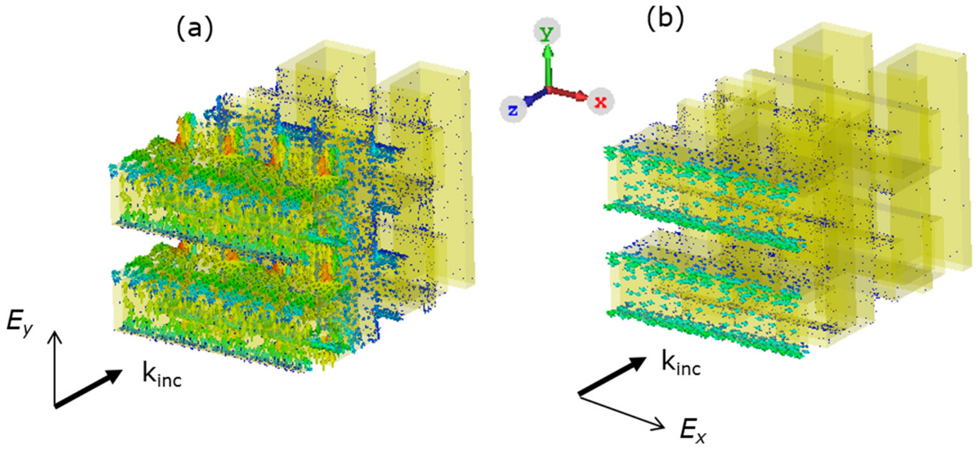

Fig. S2: Distribution of surface current density at 27 THz for (a) *y* linear polarization incidence and (b) *x* linear polarization incidence. For E_y excitation, the field penetrates and interacts with the structure, since the incident H_x field component can couple with the magnetic dipole resonance of the SCR resonators in the first/front layer. The induced current distribution then conductively couples to the other layers. In contrast, the E_x excitation cannot excite the magnetic dipole resonance; the front layer behaves in that case as a grid of uniform conducting wires, impeding the coupling of the incident field with the other (inner) SCR layers and leading to almost total reflection.

S3. Oblique incidence results for TE polarization in *xz* plane

In Fig. S3 it is demonstrated that for TE polarized incidence in the *xz* incidence plane ($\mathbf{E}=E_y\mathbf{y}$), the spectral position of the absorption peak changes with incidence angle. This is because the magnetic field component does not remain parallel to the *x* axis, in contrast to TM polarization in the *yz* plane that is discussed in the manuscript (see Fig. 4).

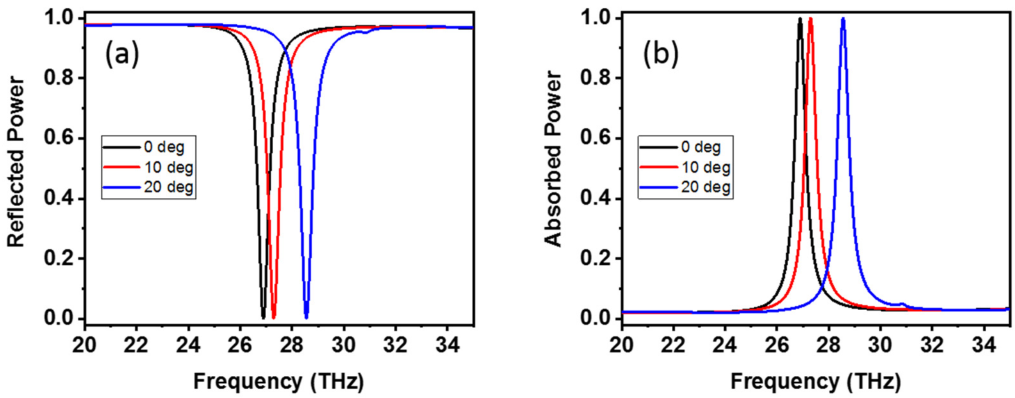

Fig. S3: Oblique incidence performance for TE polarization in the *xz* incidence plane. Reflection and absorption spectra for different incidence angles: θ = {0, 10, 20} degrees. The performance is independent of the incidence angle. The spectral position of the absorption peak changes with incidence angle, since the magnetic field component does not remain parallel to the *x* axis.

Acknowledgements

This work was partially supported by the European Union's Horizon 2020 FETOPEN programme under project VISORSURF grant agreement No. 736876, project NANOPOLY grant agreement No. 829061, and project ULTRACHIRAL grant agreement No. 737071, the project HELLAS-CH (MIS 5002735) implemented under “Action for Strengthening Research and Innovation Infrastructures”, funded by the Operational Program “Competitiveness, Entrepreneurship and Innovation” (NSRF 2014-2020) co-financed by Greece and the European Union (European Regional Development Fund), and FEMTOSURF, the European Union’s Horizon 2020 research and innovation program under grant agreement No. 825512. The authors acknowledge Dr. A. Selimis for useful discussions on sample fabrication.
